# Down-Regulation of Lnc-CYP7A1-1 Rejuvenates Aged Human Mesenchymal Stem Cells to Improve Their Efficacy for Heart Repair Through SYNE1

**DOI:** 10.3389/fcell.2020.600304

**Published:** 2020-11-19

**Authors:** Jun Dong, Jianwei Liu, Yueqiang Wen, Stephanie W. Tobin, Chongyu Zhang, Huiling Zheng, Zehan Huang, Yongtao Feng, Dongcheng Zhang, Shiming Liu, Zhenhui Zhang, Jiao Li

**Affiliations:** ^1^Guangzhou Institute of Cardiovascular Disease, Guangdong Key Laboratory of Vascular Diseases, State Key Laboratory of Respiratory Disease, The Second Affiliated Hospital, Guangzhou Medical University, Guangzhou, China; ^2^Division of Cardiovascular Surgery, Toronto General Hospital Research Institute, University Health Network, Toronto, ON, Canada; ^3^Department of Cardiovascular Surgery, The Second Affiliated Hospital, Guangzhou Medical University, Guangzhou, China; ^4^Sunnybrook Research Institute, Toronto, ON, Canada

**Keywords:** aging, rejuvenation, lncRNAs, myocardial infarction, proliferation, mesenchymal stem cell

## Abstract

**Background:**

Several long non-coding RNAs (lncRNAs) have been associated with cell senescence, termed senescence-associated lncRNAs (SAL-RNAs). However, the mechanisms involved for SAL-RNAs in aging are not fully elucidated. In the present study, we investigated the effects of SAL-RNAs on aged human bone marrow-derived mesenchymal stem cells (hBM-MSCs), and the possible means to counteract such effects to improve the regenerative capacity of aged hBM-MSCs.

**Methods:**

By comparing the lncRNAs expression of hBM-MSCs derived from young and old individuals, lnc-CYP7A1-1 was identified as being significantly increased with age. Using predictive software, the expression of Spectrin Repeat Containing Nuclear Envelope Protein 1 (SYNE1), was found to be decreased with age. Next, through lentiviral constructs, we downregulated the expression of lnc-CYP7A1-1 or SYNE1 in hBM-MSCs separately. Additionally, hBM-MSCs proliferation, survival, migration, and senescence were investigated *in vitro. In vivo*, lnc-CYP7A1-1 downregulated aged hBM-MSCs were implanted into infarcted mouse hearts after myocardial infarction (MI), and cardiac function was measured. Through lentivirus-mediated downregulation of lnc-CYP7A1-1 in aged hBM-MSCs, we revealed that cell senescence was decreased, whereas cell proliferation, migration, and survival were increased. On the other hand, downregulation of SYNE1, the target gene of lnc-CYP7A1-1, in young hBM-MSCs increased cell senescence, yet decreased cell proliferation, migration, and survival. Downregulation of lnc-CYP7A1-1 in aged hBM-MSCs induced cell rejuvenation, yet this effect was attenuated by repression of SYNE1. *In vivo*, transplantation of lnc-CYP7A1-1 downregulated old hBM-MSCs improved cardiac function after MI.

**Conclusion:**

Down-regulation of lnc-CYP7A1-1 rejuvenated aged hBM-MSCs and improved cardiac function when implanted into the infarcted mouse hearts, possibly through its target gene SYNE1.

## Introduction

Human bone marrow (hBM)-derived mesenchymal stem cells (MSCs), with their abilities of multipotent potential and promoting regenerative processes in host tissues via paracrine signaling, show great promise in tissue repairing. The potency of hBM-MSCs in the treatment of cardiovascular disease has been shown to decrease with the age of the donor (Dong et al., 2018). This decreased potency of hBM-MSCs has a significant impact on the use of autologous stem cells for treating a predominantly older cohort of patients with cardiovascular disease (Li S.-H. et al., 2013; Li et al., 2018, 2019). Part of this reduced efficacy is due to hBM-MSCs taking on a senescent phenotype and losing their proliferative capacity (Yan et al., 2017). To circumvent this deficiency of hBM-MSCs, a better understanding of the aging process, and finding means to restore the cells to a younger state, are required.

Long non-coding RNAs are non-coding RNAs greater than 200 base pairs in length. Their functions range from transcriptional to translational regulation by binding to DNA or RNA, and can also affect protein activity (Derrien et al., 2012). In humans, approximately 24% of all RNAs are lncRNA (Atianand and Fitzgerald, 2014), yet their diverse functions and incomplete/variable annotations have made them difficult to study. With respect to aging, several lncRNAs have been associated with senescence, termed senescence-associated lncRNAs (SAL-RNAs) (Abdelmohsen et al., 2013). Some examples of SAL-RNAs include ANRVIL, MALAT1, and H19 (Gomez-Verjan et al., 2018). Mechanisms for SAL-RNAs involved in aging range from transcriptional repressors/activators (Dimitrova et al., 2014; Montes et al., 2015), mRNA stability (Kumar et al., 2014), protein localization (Wu et al., 2015)/ubiquitination (Yoon et al., 2013)/translation (Abdelmohsen et al., 2014), to telomere remodeling (Cusanelli and Chartrand, 2015). For their non-coding nature and particularities, lncRNAs are emerging as potential targets for anti-aging therapies (Tan and Bird, 2016).

With age, MSCs lose proliferative potential and take on a senescent phenotype (Goodell and Rando, 2015; Ermolaeva et al., 2018). However, the role of lncRNAs in MSC aging is still an unexplored field. To better understand how lncRNAs change with aging, we compared the expression profiles of lncRNAs in young and old hBM-MSCs. Using significance analysis of lncRNA expression microarray software, we identified 12 lncRNAs, including lnc-CYP7A1-1, changed with aging. We found that lnc-CYP7A1-1 expression increased with age and postulated that it may play a role in hBM-MSC senescence. Using lentiviral knockdown of lnc-CYP7A1-1, we investigated hBM-MSC activities *in vitro*, including proliferation, survival, migration and senescence. Using predictive software, we identified a putative target gene interacting with lnc-CYP7A1-1, Spectrin Repeat Containing Nuclear Envelope Protein 1 (SYNE1), and found that its expression decreased with age. We characterized the relationship between lnc-CYP7A1-1 and SYNE1 *in vitro* using both gain and loss of function approaches. Lastly, the detrimental effect of lnc-CYP7A1-1 was investigated *in vivo.* After MI, old or lnc-CYP7A1-1 downregulated old hBM-MSCs were transplanted into the infarcted mouse hearts. Subsequently, mice heart function and scar thickness was evaluated. We found that lnc-CYP7A1-1 was increased in aged hBM-MSCs and played a role in hBM-MSC senescence. Furthermore, we proved that down-regulation of lnc-CYP7A1-1 rejuvenated aged hBM-MSCs, and improved cardiac function, when implanted into the infarcted mouse hearts, possibly through its target gene SYNE1.

## Materials and Methods

### HBM-MSCs Culture

Human BM was collected during cardiac valve replacement surgery, all the procedures were approved by the Research Ethics Board of Guangzhou Medical University and the Hospital’s Ethics Committee, and patients provided written informed consent which was approved by the Research Ethics Committee of Guangzhou Medical University. Young BM was obtained from young patients (15 females and 15 males, 23.4 ± 3.9 years), and old BM from old patients (15 females and 15 males, 72 ± 4.5 years). All patients were of the same pathological status, and received the same medical treatments, with no genetic diseases or malignancies based on the primary diagnosis were used.

The hBM-MSCs were cultured as previously described (Dong et al., 2018). Briefly, after centrifugation through a Ficoll-Paque gradient (1.077 g/mL density; GE Healthcare, Kretztechnik, Zipf, Austria), cells were separated and plated in IMDM medium supplemented with 100 U/ml penicillin, 100 μg/ml streptomycin, and 10% fetal bovine serum (FBS). After 48 h, non-adhesive cells were removed by changing the culture medium. The adhesive cells were harvested for passage when confluence reached approximately 80%.

### Cell Proliferation Assay

Human bone marrow-derived mesenchymal stem cells were cultured from different group BM and characterized as previously (Dong et al., 2018). For BrdU (5-bromo-2′-deoxyuridine, Sigma, Cat#: B5002) labeling, cells (2 × 10^4^/cm^2^) were seeded with BrdU supplementary (10 μM/mL). After BrdU pulse chasing for 72 h in hypoxic conditions (0.1% O_2_), the cells were fixed for immunofluorescent staining with BrdU antibody (Abcam, Cat#: ab6326). An MTT assay was also used to detect viable proliferating cells at 1, 3, 5, and 7 days after plating. In each well (4,000 cells/well), 50 μl of 1 mg/ml solution of MTT in PBS was added for 4 h on each of the cells. The results were measured at 560 nm test and 690 nm reference wavelength by using an automatic plate reader.

### Cell Survival Evaluation

Human bone marrow-derived mesenchymal stem cells (1 × 10^4^/cm^2^) were cultured for 72 h under hypoxic conditions. Cell survival rate was measured by the Cell Counting Kit-8 (CCK8; Dojindo, Kumamoto, Japan).

### Cell Migration Assay

The trans-well assay was used to study hBM-MSCs migration. Briefly, cells were harvested and plated (1 × 10^4^/cm^2^) in a trans-well cell culture insert (8-μm diameter pores). After 24 h in hypoxic conditions, cells that migrated to the other side of the membrane were fixed and stained. The wound-scratch assay was also used to study hBM-MSC migration (2 × 10^4^/cm^2^). Scratches were created with a p200 pipette tip. After 12 h in hypoxic conditions, images were obtained using a microscope (Nikon Eclipse Ti) after washing.

### SA-β-Gal Staining

Human bone marrow-derived mesenchymal stem cells (2 × 10^4^/cm^2^) were stained with the senescence β-galactosidase staining kit (Cell Signaling, Cat#: 9860). Pictures were taken using a Nikon microscope.

### Real-Time Reverse Transcription-Polymerase Chain Reaction

The expression of senescence-related genes (p16INK4a and p27Kip1) and lncRNAs were evaluated using real-time reverse transcription-polymerase chain reaction. GAPDH was used as a housekeeping gene. Real-time polymerase chain reaction was conducted using SensiFAST SYBR Green PCR Master Mix (Bioline USA Inc., Taunton, MA, United States), with the following parameters: 95°C 2 min; [95°C 5 s; 60°C 30 s for 40 cycles]. The oligonucleotide primer sequences are shown in Supplementary Table S1.

### Western Blotting

For Western blotting, 50 μg of lysate was fractionated and transferred to a PVDF membrane. The blots were reacted with the antibodies (p16INK4a [Abcam, Cat#: ab54210], p27Kip1 [Abcam, Cat#: ab32034]) overnight at 4°C. And then were incubated with horseradish peroxidase-conjugated secondary antibody at room temperature. For quantification, the density of the target bands were divided by the corresponding densitometry of β-tublin band.

### Microarray and Computational Analysis

Total RNA was isolated from hBM-MSCs after cultured in hypoxic conditions for 72 h. 12 × 135K lncRNA Expression Microarray (Arraystar, Rockville, MD, United States) was used to detected hBM-MSCs cDNA. After hybridization, the processed slides were scanned by the Axon GenePix 4000B microarray scanner. NimbleScan software was used to extract the raw data. And NimbleScan software’s implementation of RMA offered quantile normalization and background correction as previous (Jia et al., 2019). Differentially expressed genes were measured by the random variance model (Barter et al., 2017). A *p*-value was calculated by the paired *t*-test. The threshold set for up-regulated and down-regulated genes were fold change ≥ 2.0 and *p*-value ≤ 0.05. Cluster Tree-view software was used to perform hierarchical clustering, based on differentially expressed mRNAs and lncRNAs. Gene co-expression networks were used to identify interactions among genes. According to the normalized signal intensity of specific expressed genes, gene co-expression networks were built as previous (Bianchessi et al., 2015). The network adjacency was constructed between two genes, i and j, defined as a power of the Pearson correlation between the corresponding gene expression profiles xi and xj. The adjacency matrix M (i, j) was obtained and visualized as a graph, and the topological properties of this graph were measured. Only the strongest correlations (0.99 or greater) were drawn in these renderings (Li J. et al., 2013).

### Lentiviral Vector Transduction

Lentiviral constructs for inhibition of lnc-CYP7A1 or SYNE1 in hBM-MSCs were ordered from OBio Co. (Shanghai, China). The sh-CYP7A1 sequence was GCAGTTCTTAGATTCCCTTTG. The sh-SYNE1 sequence was GCTGAAGTCTTGGATCATTAA.

### Myocardial Infarction and Mice Heart Function Measurement

All animal procedures were approved by the Animal Care Committee of the Guangzhou Medical University. All experiments were carried out in accordance with the Guide for the Care and Use of Laboratory Animals (NIH, 8th Edition, 2011). Mice with infarct sizes between 30–35% of the left ventricular free wall were used in the following experiments (*n* = 20/group). HBM-MSCs (3 × 10^5^ in 20 μl serum-free IMDM medium/mouse) were transplanted into three sites around the border zone immediately after MI. Serum-free IMDM medium was injected into the border zone as a negative control. Echocardiography was used to measure the mice heart function at different time points. The mice heart scar area and thickness were measured by planimetry. Cyclosporine A (5 mg/kg) was used to induce immunosuppression during the experiments as previous (Li S.-H. et al., 2013; Li et al., 2018).

### Statistical Analysis

All values are expressed as mean SD. Analyses were performed using GraphPad InStat software (La Jolla, CA, United States). Student’s *t*-test was used for two-group comparisons. Comparisons of parameters among three or more groups were analyzed using one-way analysis of variance (ANOVA), followed by Tukey, or two-way ANOVA with repeated measures over time, which were succeeded by Bonferroni *post hoc* tests for multiple comparisons. Differences were considered statistically significant at *P* < 0.05.

## Results

### The Proliferative and Migratory Functions of hBM-MSCs Were Decreased With Aging

**FIGURE 1:**
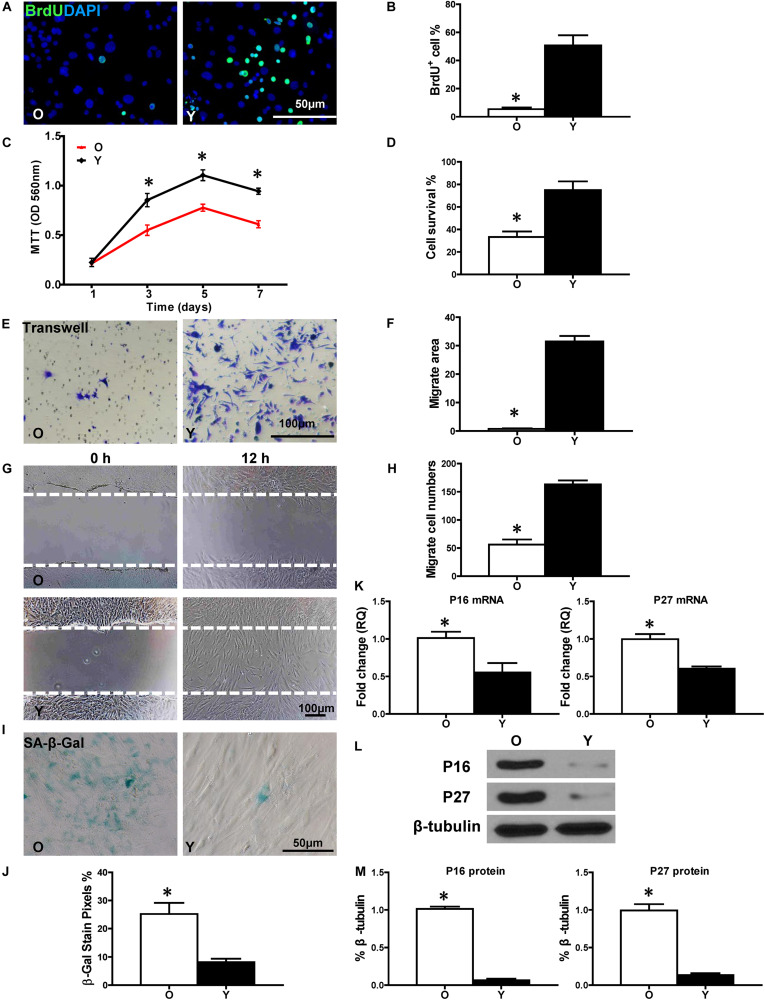
Increased cell senescence and decreased regenerative function in aged hBM-MSCs. The cell regenerative function of old (O) and young (Y) hBM-MSCs was compared. **(A)** Immunofluorescence staining of BrdU and **(B)** quantification of BrdU^+^ (proliferating cells) cells in the O and Y hBM-MSCs. **(C)** Cell proliferation was determined by the MTT assay. **(D)** Cell survival was evaluated in O and Y hBM-MSCs. Cell migration was evaluated by the trans-well **(E,F)** and wound-scratch **(G,H)** assays. SA-β-Gal staining and quantification of cell senescence in O and Y hBM-MSCs **(I,J)**. The expression of senescence-related genes **(K)** and proteins of p16^INK4a^ and p27^Kip1^ **(L,M)** in O and Y hBM-MSCs. *n* = 6/group for all the assays; ^∗^*P* < 0.05 O vs. Y.

First, BrdU pulse-chasing was used to evaluate cell proliferation in old (O) and young (Y) hBM-MSCs after isolation and culturing. When compared to Y hBM-MSCs, the percentage of BrdU^+^ cells was significantly lower in O hBM-MSCs (Figures 1A,B). The same trend was found by the MTT assay, showing decreased cell proliferative activity in O hBM-MSCs (Figure 1C). The cell survival was decreased in O compared with Y hBM-MSCs when evaluated by CCK-8 assay (Figure 1D). Next, cell migration was detected by transwell and wound-scratch assays (Figures 1E–H), which both showed significantly lower migration rates (Figures 1F,H) in O compared to YhBM-MSCs. Senescence-associated beta galactosidase (SA-β-Gal) staining revealed more positive cells in O than Y hBM-MSCs (Figures 1I,J). Accordingly, the expression of senescence-related genes, p16^INK4a^ and p27^Kip1^, were significantly increased (Figure 1K) in O than in Y hBM-MSCs. This finding was confirmed by Western blots, showing increased protein expression of p16^INK4a^ and p27^Kip1^ in O relative to Y hBM-MSCs (Figures 1L,M). All these results pointed out the decreased proliferative and migratory abilities, and increased cell senescence, in O compared to Y hBM-MSCs.

### Expression Profiles of lncRNAs in Y and O hBM-MSCs

**FIGURE 2:**
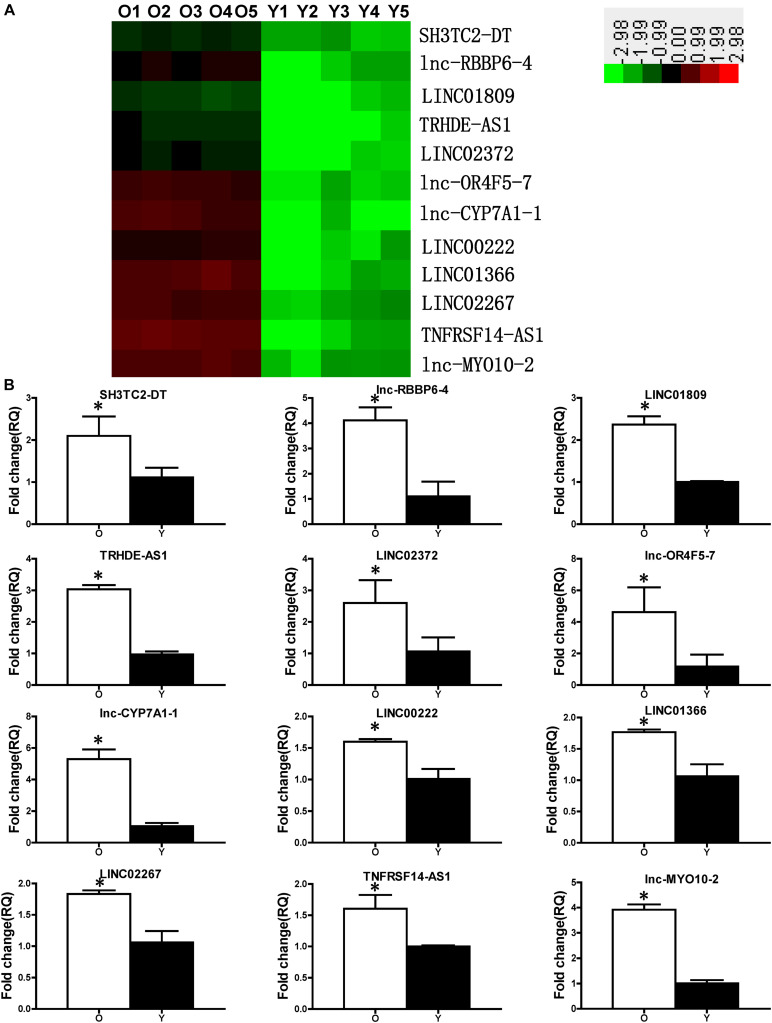
Expression profile of lncRNAs in young and old hBM-MSCs. **(A)** lncRNA expression in old (O) and young (Y) hBM-MSCs was determined by microarray analysis. **(B)** Differential lncRNA expression was validated by real-time qPCR. *n* = 5/group; ^∗^*P* < 0.05 O vs. Y.

Microarrays were carried out to profile the expression of different lncRNAs and mRNAs in Y and O hBM-MSCs. Twelve lncRNAs were identified as significantly increased in O compared with that of Y hBM-MSCs, by using significance analysis of microarray software (Figure 2A). Next, real-time qPCR was used to validate the microarray data. Notably, SH3TC2-DT, lnc-RBBP6-4, LINC01809, TRHDE-AS1, LINC02372, lnc-OR4F5-7, lnc-CYP7A1-1, LINC00222, LINC01366, LINC02267, TNFRSF14-AS1 and lnc-MYO10-2 were significantly increased in O compared to Y hBM-MSCs (Figure 2B). Furthermore, lnc-CYP7A1-1 showed the most dramatic increase with a 5.29-fold increase in O, compared to Y hBM-MSCs, suggesting it may play an active role in cell aging and senescence.

### Down-Regulation of lnc-CYP7A1-1 in O hBM-MSCs Restored Cell Regenerative Functions

**FIGURE 3:**
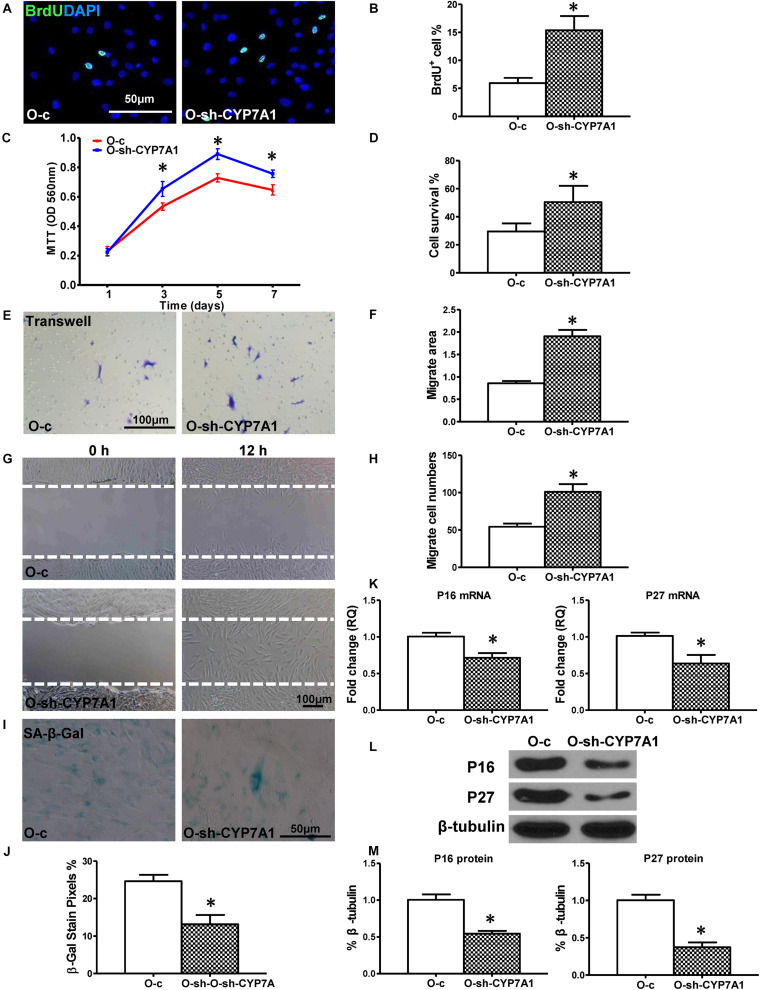
Down-regulation of lnc-CYP7A1-1 in old hBM-MSCs restored cell regenerative function and decreased senescence. Old (O) hBM-MSCs were transfected with inhibition lentivirus (O-sh-CYP7A1) or control lentivirus (O-c), respectively. Cell regenerative function was compared. **(A)** Immunofluorescent staining of BrdU and **(B)** quantification of BrdU^+^ (proliferating) cells in the hBM-MSCs. **(C)** Cell proliferation was determined by the MTT assay. Cell survival was evaluated in hBM-MSCs **(D)**. Cell migration was evaluated by the trans-well **(E,F)** and wound scratch **(G,H)** assays. SA-β-Gal staining and quantification of cell senescence in hBM-MSCs **(I,J)**. The expression of senescence-related genes **(K)** and proteins of p16^INK4a^ and p27^Kip1^ **(L,M)** in hBM-MSCs. *n* = 6/group for all the assays; ^∗^*P* < 0.05 O-sh-CYP7A1 vs. O-c.

Next, to investigate whether down-regulation of lnc-CYP7A1-1 can restore some aspects of regenerative function in O hBM-MSCs, a lentiviral construct was produced (sh-CYP7A1) to inhibit lnc-CYP7A1-1 expression in O hMSCs (O-sh-CYP7A1, Supplementary Figure S1). In accordance with our expectations, cell proliferation was increased in O-sh-CYP7A1 when compared to control lentivirus-transduced O hMSCs (O-c). The percentage of BrdU^+^ cells was much higher in O-sh-CYP7A1, compared to control O-c (Figures 3A,B), and the same trend was also observed by the MTT assay (Figure 3C). Next, the cell survival was increased in O-sh-CYP7A, compared to O-c, in the CCK-8 assay (Figure 3D). Cell migration, when detected by trans-well and wound-scratch assays (Figures 3E–H), was significantly higher in O-sh-CYP7A1 compared to O-c. SA-β-Gal staining revealed fewer positive cells in O-sh-CYP7A1 than O-c (Figures 3I,J). The mRNA expression of p16^INK4a^ and p27^Kip1^ was significantly decreased (Figure 3K) in O-sh-CYP7A1 than O-c, and this was confirmed by Western blots showing decreased protein expression of p16^INK4a^ and p27^Kip1^ in O-sh-CYP7A1, relative to O-c (Figures 3L,M). These results revealed that inhibition of lnc-CYP7A1-1 restored the regenerative capacity, and decreased cell senescence, in O hBM-MSCs.

### The Expression of SYNE1 Was Inhibited by lnc-CYP7A1-1

**FIGURE 4:**
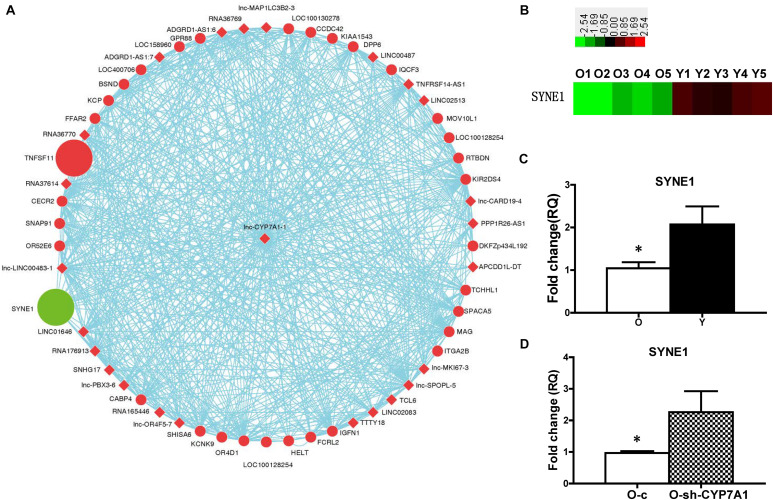
The expression of SYNE1 was inhibited by lnc-CYP7A1-1. **(A)** Gene co-expression networks were built to detect the interactions among lncRNAs and genes. Red color indicates expression up-regulated and green color indicates expression down-regulated. The dots represents genes and the diamond represents lncRNAs. **(B)** The expression of SYNE1 in old (O) and young (Y) hBM-MSCs was determined by microarray analysis. **(C)** The expression of SYNE1 in Old (O) and young (Y) hBM-MSCs was compared by real-time qPCR. **(D)** Old (O) hBM-MSCs were transfected by inhibition lentivirus (O-sh-CYP7A1) or control lentivirus (O-c), respectively. The expression of SYNE1 was compared by real-time qPCR. *n* = 5/group; *P < 0.05 O vs. Y, O-c vs. O-sh-CYP7A1.

Gene co-expression network was built to detect the interactions among lncRNA and genes (Figure 4A). SYNE1 was identified as a strong candidate to interact with lncRNA CYP7A1-1. SYNE1 (aka Nesp-1) is a component of the LINC (Linker of Nucleoskeleton and Cytoskeleton) complex, which connects the nuclear envelope to the cytoskeleton. The expression of SYNE1 was significantly decreased in O compared to Y hBM-MSCs, in contrast to the increase of lnc-CYP7A1-1 at O hBM-MSCs (Figure 4B). The down-regulation of SYNE1 in O hBM-MSCs was confirmed by real-time qPCR (Figure 4C). On the other hand, compared to control lentiviral transduced O-c, the expression of SYNE1 was significantly increased when lnc-CYP7A1-1 was down-regulated in O-sh-CYP7A1 hBM-MSCs (Figure 4D), strongly suggesting SYNE1 as the underlying target gene of lnc-CYP7A1-1.

### Down-Regulation of SYNE1 in Y hBM-MSCs Decreased Cell Regenerative Functions

**FIGURE 5:**
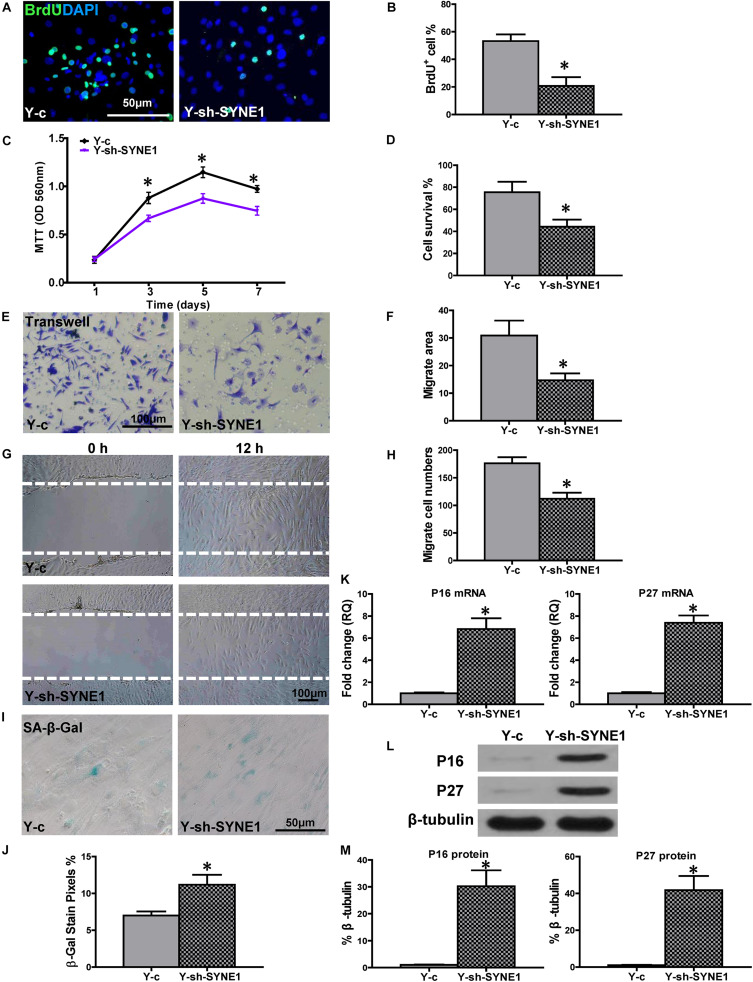
Down-regulation of SYNE1 in Y hBM-MSCs decreased cell regenerative functions. Young (Y) hBM-MSCs were transfected by inhibition lentivirus (Y-sh-SYNE1) or control lentivirus (Y-c), respectively, and cell regenerative functions were compared. **(A)** Immunofluorescent staining of BrdU and **(B)** quantification of BrdU^+^ (proliferating) cells in the hBM-MSCs. **(C)** Cell proliferation was determined by the MTT assay. Cell survival was evaluated in hBM-MSCs **(D)**. Cell migration was evaluated by the transwell **(E,F)** and wound-scratch **(G,H)** assays. SA-β-Gal staining and quantification of cell senescence in hBM-MSCs **(I,J)**. The expression of senescence-related genes **(K)** and proteins of p16^INK4a^ and p27^Kip1^ in hBM-MSCs **(L,M)**. *n* = 6/group for all the assays; *P < 0.05 Y-sh-SYNE1 vs. Y-c.

To further explore the role of SYNE1, relative to cell regenerative capacity, a lentiviral construct was produced (sh-SYNE1) to inhibit SYNE1 expression in Y hBM-MSCs (Y-sh-SYNE1, Supplementary Figure S2). Cell proliferation was examined in Y-sh-SYNE1, and compared to control lentivirus transduced Y hMSCs (Y-c). The percentage of BrdU^+^ cells was much lower in Y-sh-SYNE1, compared to control Y-c (Figures 5A,B), and similar result was observed by MTT assay (Figure 5C). Next, cell survival was decreased in Y-sh-SYNE1, compared to Y-c, in the CCK-8 assay (Figure 5D). Cell migration, again detected by trans-well and wound-scratch assays (Figures 5E–H), was significantly lower in Y-sh-SYNE1 than Y-c. SA-β-Gal staining revealed more positive cells in Y-sh-SYNE1 than Y-c (Figures 5I,J). The mRNA expression of p16^INK4a^ and p27^Kip1^ was significantly increased (Figure 5K) in Y-sh-SYNE1 than Y-c. Western blots confirmed increased p16^INK4a^ and p27^Kip1^ protein expression in Y-sh-SYNE1, relative to Y-c (Figures 5L,M). All these findings suggested SYNE1 as an important factor involved in cell regeneration.

### Down-Regulation of SYNE1 in O-sh-CYP7A1 hBM-MSCs Reduced Cell Regenerative Ability

**FIGURE 6:**
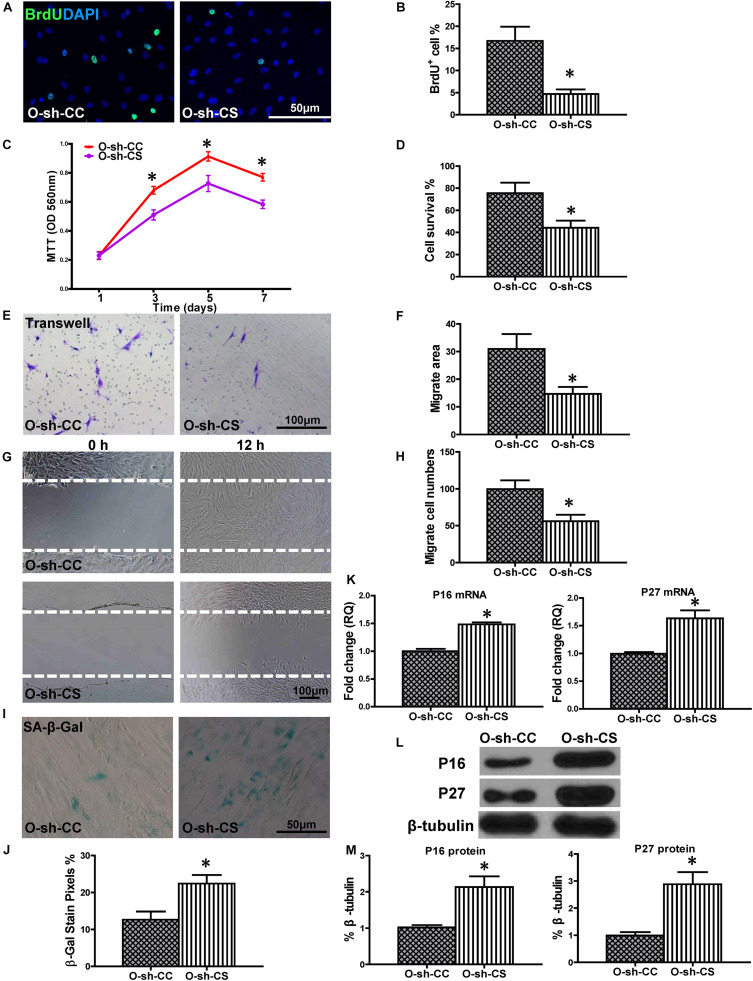
Down-regulation of SYNE1 in O-sh-CYP7A1 hBM-MSCs reduced cell regenerative ability. O-sh-CYP7A1 hBM-MSCs were transfected by SYNE1 inhibition lentivirus (O-sh-CS) or control lentivirus (O-sh-CC), respectively, and cell regenerative functions were compared. **(A)** Immunofluorescent staining of BrdU and **(B)** quantification of BrdU^+^ (proliferating cells) cells in the hBM-MSCs. **(C)** Cell proliferation was determined by the MTT assay. Cell survival was evaluated in the hBM-MSCs **(D)**. Cell migration was evaluated by the transwell **(E,F)** and wound scratch **(G,H)** assays. SA-β-Gal staining and quantification of cell senescence in hBM-MSCs **(I,J)**. The expression of senescence-related genes **(K)** and proteins of p16^INK4a^ and p27^Kip1^ in the hBM-MSCs **(L,M)**. *n* = 6/group; *P < 0.05 O-sh-CS vs. O-sh-CC.

To confirm the causative relationship between lnc-CYP7A1-1 and SYNE1, the expression of SYNE1 was inhibited in O-sh-CYP7A1 hBM-MSCs by transduction with the sh-SYNE1 lentivirus (O-sh-CS, Supplementary Figure S3). Cell proliferation was assessed in O-sh-CS and compared to negative control lentivirus transfected O-sh-CYP7A1 hBM-MSCs (O-sh-CC). The percentage of BrdU^+^ cells was lower in O-sh-CS compared to O-sh-CC (Figures 6A,B). This result was confirmed by the MTT assay (Figure 6C). Next, cell survival was decreased in O-sh-CS, compared with O-sh-CC, in the CCK-8 assay (Figure 6D). Cell migration was evaluated by trans-well and wound-scratch assays (Figures 6E–H), and was found to be significantly lower in O-sh-CS, compared to O-sh-CC. SA-β-Gal staining revealed more positive cells in O-sh-CS than in O-sh-CC (Figures 6I,J). The p16^INK4a^ and p27^Kip1^ mRNA expression was significantly increased (Figure 6K) in O-sh-CS than in O-sh-CC, and this result was further confirmed at the protein level by Western blot (Figures 6L,M). All these findings strengthened our hypothesis that SYNE1 is the underlying target gene of lnc-CYP7A1-1.

### Implantation of lnc-CYP7A1-1 Downregulated O hBM-MSCs Into Infarcted Mouse Hearts Improved Heart Function After MI

**FIGURE 7:**
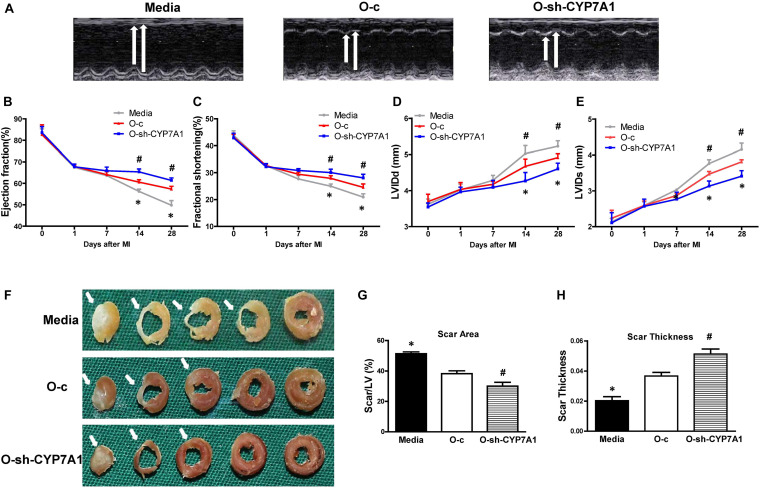
Implantation of lnc-CYP7A1-1 downregulated old hBM-MSCs into infarcted mouse hearts improved cardiac function after MI. Cardiac function was determined by echocardiography in mice which received implantations of control medium (Media), control vector-transfected old hBM-MSCs (O-c), or lnc-CYP7A1-inhibited old hBM-MSCs (O-sh-CYP7A1) into the border region immediately following myocardial infarction (MI). Cardiac function measured by echocardiography, before (0 day), as well as 1, 7, 14, and 28 days after MI. **(A)** Representative M-mode echocardiographic images. **(B)** Ejection fraction. **(C)** Fractional shortening. **(D)** Left ventricular internal end-diastolic dimension (LVIDd). **(E)** Left ventricular internal end-systolic dimension (LVIDs). **(F)** Representative whole sectioned heart (from base to apex) at 28 days after MI to show scar areas (arrows) **(G)** and scar size thickness **(H)**. *n* = 6/group for all the experiments. *P < 0.05, Media vs. other groups; ^#^*P* < 0.05, O-sh-CYP7A1 vs. O-c.

To evaluate whether down-regulation of lnc-CYP7A1-1 levels in O hBM-MSCs can maximize the beneficial effects of stem cell therapy, CYP7A1-1-downregulated O hBM-MSCs (O-sh-CYP7A1) were implanted into infarcted mouse hearts. Heart function was measured by echocardiography in mice which received implantation of control medium (Media), control vector-transfected O hBM-MSCs (O-c), or CYP7A1-1 downregulated O hBM-MSCs (O-sh-CYP7A1), into the border region immediately after MI. Heart function was evaluated before MI (0 days), as well as 1, 7, 14, and 28 days after MI. Representative M-mode echocardiographic images was taken 28 days post MI (Figure 7A). After MI, there was a significant decrease in ejection fraction (EF; Figure 7B) and fractional shortening (FS; Figure 7C), along with an increase in left ventricular internal end-diastolic dimension (LVIDd; Figure 7D) and left ventricular internal end-systolic dimension (LVIDs; Figure 7E), in all three groups. However, there was an improvement in all of these parameters in the O-sh-CYP7A1 group when compared with O-c and media groups (Figures 7B–E). Similarly, the infarct size at 28 days post MI was smaller (Figures 7F,G), and the scar thickness (Figure 7H) larger in O-sh-CYP7A1, when compared with the O-c and the media groups. All evidence, therefore, indicated that the down-regulation of lnc-CYP7A1-1 enhanced the therapeutic efficacy of O hBM-MSCs and effectively improved heart function. The survival of implanted lnc-CYP7A1-1-downregulated old hBM-MSCs was also evaluated through lentiviral-mediated GFP expression in the border region of the mouse hearts at 3 days post MI. In agreement with *in vitro* data, downregulation of lnc-CYP7A1-1 expression (O-sh-CYP7A1) increased implanted cell survival when compared with the group receiving O-c hBM-MSCs (Supplementary Figure S4).

### Down-Regulation of lnc-CYP7A1-1 in O hBM-MSCs Changed the Cell Paracrine Function *in vitro*

To investigate whether down-regulation of lnc-CYP7A1-1 can change the cell paracrine function. Control vector-transfected O hBM-MSCs (O-c) or CYP7A1-1 downregulated O hBM-MSCs (O-sh-CYP7A1) were cultured in serum-free medium under hypoxic conditions (0.1% O_2_) for 72 h. The mRNA expression of vascular endothelial growth factor A (VEGFA), platelet derived growth factor A (PDGFA), fibroblast growth factor 2 (FGF2), insulin-like growth factor 1 (IGF1), transforming growth factor beta-1 (TGFB1), angiogenin (ANG), and C-C motif chemokine ligand 2 (CCL2) was quantified by RT-qPCR. The mRNA expression of VEGFA, PDGFA, and FGF2 was increased in O-sh-CYP7A1 when compared to O-c group (Supplementary Figure S5). These results revealed that inhibition of lnc-CYP7A1-1 may increase the cell paracrine function in O hBM-MSCs.

## Discussion

In this study, we found that lnc-CYP7A1-1 contributed to hBM-MSCs senescence, as the evidence showed that increased lnc-CYP7A1-1 expression in old hBM-MSCs was associated with decreased cell proliferative ability, survival, and migratory ability, along with increased senescence and senescence-related gene expression. In contrast, down-regulation of lnc-CYP7A1-1 improved regenerative capacities, and decreased cell senescence, in old hBM-MSCs. Based on predictive software, we characterized a putative lnc-CYP7A1-1 interacting gene, SYNE1, whose expression was decreased in old hBM-MSCs. Through inhibiting SYNE1 expression in young hBM-MSCs, using a lentiviral construct, we confirmed the role of SYNE1 as an important factor involved in cell regeneration. Furthermore, via down-regulation of SYNE1 expression in O-sh-CYP7A1 hBM-MSCs, we established the causative relationship between lnc-CYP7A1-1 and SYNE1, showing that inhibition of SYNE1 reversed the beneficial effects stemming from lnc-CYP7A1-1 down-regulation in old hBM-MSCs. *In vivo* implantation of lnc-CYP7A1-1 downregulated old hBM-MSCs into infarcted mouse hearts improved cardiac function after MI, suggesting that down-regulation of lnc-CYP7A1-1 enhanced the therapeutic efficacy of old hBM-MSCs for cardiac repair.

Long non-coding RNAs have been reported as an unexploited reservoir of potential therapeutic targets for reprogramming cell function and aging. Huang et al. (2015) found that lncRNA H19 promotes osteogenic differentiation of hMSCs, and miR-675 partially mediates lncRNA H19-induced pro-osteogenic activity. The expression of TGF-b1, HDAC4/5 and Smad3 phosphorylation was decreased, but the expression of osteogenic markers was increased by H19/miR-675 (Huang et al., 2015). LINC00707 has also been proved to directly bind to miR-370-3p and promote osteogenic differentiation in hMSCs (Jia et al., 2019). Studies have reported that lncRNA ROCR increases during chondrogenic differentiation of hMSCs; the lncRNA is involved in inducing SOX9 gene and cartilage gene expression there (Barter et al., 2017). The role of lncRNAs has also been studied in other cell types. Compared to early passage fibroblasts (young), late-passage fibroblasts (old) have decreased expression of SAL-RNA1. Furthermore, inhibiting the expression of SAL-RNA1 in fibroblasts increases cell senescence (Abdelmohsen et al., 2013). Another study in fibroblasts showed that Zeb2-NAT lncRNA has higher expression levels in old fibroblast cells, and modulation of its expression can improve pluripotent cell reprogramming from old fibroblasts (Bernardes de Jesus et al., 2018). In aging and senescent endothelial cells, ASncmtRNA-2 accumulates at the G2/M phase of cell cycle, and causes cell aging by inducing hsa-miR-4485 and hsa-miR-1973 expression (Bianchessi et al., 2015). In agreement with our findings, all pieces of evidence point toward the important role of lncRNAs for reprogramming cell function and aging.

In our previous studies, we have demonstrated that with aging, proliferation and differentiation capacity decreased, and cell senescence increased, in hBM-MSCs (Li J. et al., 2013; Dong et al., 2018). Effective strategies to rejuvenate aged hBM-MSCs to improve their regenerative capability are required to maximize the beneficial effects of stem cell therapy, such as the small RNAs (Liu et al., 2019). By bone marrow reconstitution animal model, we also demonstrated that young bone marrow Scal-1 cells can rejuvenate age animal heart function after MI. Examination of the underlying molecular mechanisms revealed that young bone marrow Scal-1 cells secreted more growth factors, such as Tgfβ1 and Cxcl12, in order to regenerate the aged heart (Li et al., 2018, 2019). In this study, we also detected paracrine functional changes, with down-regulation of lnc-CYP7A1-1 in O hBM-MSCs. The mRNA expression of VEGFA, PDGFA, and FGF2 was increased in O-sh-CYP7A1 when compared to O-c groups. These results revealed that inhibition of lnc-CYP7A1-1 may potentiate paracrine functions in O hBM-MSCs. In the present study, we identified target lncRNAs associated with MSCs aging. These aging related lncRNAs may modulate the regenerative abilities of hBM-MSCs. Indeed, we specifically identified lncRNA CYP7A1-1, as it had the most dramatic increase in expression, and showed the most prominent effects in aged hBM-MSCs. Lnc-CYP7A1-1 is an intergenic lncRNA, located on human Chromosome 8 (hg38 chr8:58258605-58272587). We found that suppression of lnc-CYP7A1-1 expression improved proliferation, cell survival, migration, and paracrine function along with reducing senescence in old hBM-MSCs, which is consistent with the idea that lncRNA contributes to the pathological phenotypes associated with hBM-MSCs (Barter et al., 2017).

A portion of the difficulties for studying lncRNAs in a biological context is in determining their underlying mediators. Using predictive software, we found a putative interacting partner of lnc-CYP7A1-1, SYNE1 (Nesprin-1). SYNE1 is a structural protein that links the nucleus to the cytoskeleton. It plays a role in cardiomyocyte and skeletal muscle development, particularly with respect to the DNA damage response pathway (Razafsky and Hodzic, 2015). In Emery–Dreifuss muscular dystrophy and dilated cardiomyopathy patients, mutations in SYNE1 have been found, which affects nuclear morphology and impairs protein-protein interaction with lamin A/C and SUN2 (Meinke et al., 2014). Defection in myoblast differentiation and fusion are observed when expressing SYNE1 mutants in C2C12 cells (Zhou et al., 2017). SYNE1 and SYNE2 have also been reported to play key roles in neurogenesis and neuronal migration in mice (Zhang et al., 2009). In human umbilical vein endothelial cells, cell migration and endothelial loop formation capacity is decreased when either SYNE1 or SYNE2 is suppressed (King et al., 2014). The modulation of SYNE1 expression also impacted stem cell pluripotency and differentiation capacity (Smith et al., 2011; Yang et al., 2015). Furthermore, SYNE1 has been reported to participate in laminopathies and lamin-associated signaling pathways, leading to laminopathies and premature aging (Zhavoronkov et al., 2012). Most importantly, inhibiting the expression of SYNE1 in rat MSCs decreases cell proliferation and increases apoptosis (Yang et al., 2013). In the present study, we also found that SYNE1 had a beneficial effect on BM-MSC function, as its loss in young BM-MSCs reduced their proliferative capacity. Interestingly, lnc-CYP7A1-1 and SYNE1 show inverse expression patterns during aging, where SYNE1 was abundant in young BM-MSCs, while lnc-CYP7A1-1 was highly expressed in old BM-MSCs. Loss of lnc-CYP7A1-1 in old BM-MSCs increased SYNE1 expression, implying possible negative regulation of SYNE1 by lnc-CYP7A1-1. Additionally, the beneficial effects of lnc-CYP7A1-1 knockdown on old BM-MSCs were lost when SYNE1 expression was also reduced, suggesting that lnc-CYP7A1-1 and SYNE1 act on a shared signaling pathway in modulating BM-MSC function. All these findings supported our notion that SYNE1 play an important role in mediating cell function and regeneration, and may be the key downstream mediator of lnc-CYP7A1-1. Recent studies have found that, as pseudogenes, lncRNAs can act as miRNA “sponges” by sharing common microRNA recognition elements (MREs), thereby inhibiting normal miRNA activity (Yang et al., 2018). In a study related to hMSCs, Jia et al. have reported that LINC00707 effectively inhibits miR-370-3p to promote osteogenesis, in which it serves as a competing endogenous RNA for the target gene of miR-370-3p, WNT2B. By directly binding miR-370-3p, LINC00707 upregulates WNT2B expression (Jia et al., 2019). In a study to evaluate the chondrogenic differentiation of hMSCs, lncRNA ADAMTS9-AS2 has been reported to serve as a competing endogenous RNA for miR-942-5p, which is involved in regulating the expression of Scrg1, a transcription factor promoting chondrogenic gene expression. There, lncRNA ADAMTS9-AS2 controls hMSC chondrogenic differentiation (Huang et al., 2019). We postulate that lnc-CYP7A1-1 may act in a similar fashion in competing with microRNAs to regulate SYNE1 and hBM-MSCs functions. In our on-going study, via the MicroRNA Target Prediction Database, we found that miR-144, miR-597, and miR-421 may interact with lnc-CYP7A1-1, which may play a role in regulating SYNE1.

Mechanistically, based on our *in vitro* data showing increased cell proliferative and migratory abilities, we postulate that downregulation of lnc-CYP7A1-1 in old hBM-MSCs may improve cell survival, thus increasing the regenerative capacities of old hBM-MSCs after transplantation into infarcted mouse hearts. Indeed, we found that in agreement with our *in vitro* data, downregulation of lnc-CYP7A1-1 expression (O-sh-CYP7A1) increased implanted cell survival when compared with the group receiving O-c hBM-MSCs. Furthermore, we found that downregulation of lnc-CYP7A1-1 (O-sh-CYP7A1) increased expression of VEGFA, PDGFA, and FGF2 when compared with the O-c group, suggesting greater angiogenic potentials among these cells. In the present study, we focused on the discovery of this senescence-associated lncRNA (lnc-CYP7A1-1) and the cardio-protection effects after its downregulation in old hBM-MSCs. In our future studies, we will dissect the detailed cardioprotective mechanisms associated with the downregulation of lnc-CYP7A1-1 in old hBM-MSCs *in vivo*. We will further evaluate the survival of lnc-CYP7A1-1-downregulated old hBM-MSCs and its paracrine and angiogenic potentials *in vivo*.

## Conclusion

In summary, we have proved that the lncRNA lnc-CYP7A1-1 contributed to hBM-MSCs senescence, as the evidence showed that increased lnc-CYP7A1-1 expression in old hBM-MSCs were associated with decreased cell proliferative ability, cell survival and migratory ability, as well as increased senescence and the condition’s associated gene expression. Downregulation of lnc-CYP7A1-1 improved cell regenerative capacities and decreased cell senescence in old hBM-MSCs, probably through upregulation of its target gene SYNE1. *In vivo* implantation of lnc-CYP7A1-1-downregulated old hBM-MSCs into infarcted mouse hearts improved cardiac function after MI, suggesting that down-regulation of lnc-CYP7A1-1 rejuvenated old hBM-MSCs and improved their regenerative capability for cardiac repair. Modulation of lnc-CYP7A1-1 levels may offer a useful therapeutic intervention to maximize the efficacy of stem cell therapy.

## Data Availability Statement

The original contributions presented in the study are included in the article/Supplementary Material, further inquiries can be directed to the corresponding authors.

## Ethics Statement

The studies involving human participants were reviewed and approved by Research Ethics Committee of Guangzhou Medical University. The patients/participants provided their written informed consent to participate in this study. The animal study was reviewed and approved by Research Ethics Committee of Guangzhou Medical University.

## Author Contributions

JD, JWL, and YW contributed to analysis and interpretation of the data. ST, CZ, HZ, ZH, YF, and DZ collected and analyzed the *in vivo* data. SL, ZZ, and JL designed the study and wrote the manuscript. All authors contributed to the article and approved the submitted version.

## Conflict of Interest

The authors declare that the research was conducted in the absence of any commercial or financial relationships that could be construed as a potential conflict of interest.

## Supplementary Material

Supplementary Figure 1 The expression of lnc-CYP7A1-1 was inhibited by lentivirus. **(A)** To inhibit lnc-CYP7A1-1 expression in Old (O) hBM-MSCs, a lentiviral construct was produced (O-sh-CYP7A1). **(B)** The down-regulation of lnc-CYP7A1-1 was confirmed by real-time qPCR. *n* = 5/group; **P* < 0.05 O-sh-CYP7A1 vs. O-c. O-c: control lentivirus transduced O hBM-MSCs.

Supplementary Figure 2 The expression of SYNE1 was inhibited by lentivirus in Y hBM-MSCs. **(A)** To inhibit SYNE1 expression in young (Y) hBM-MSCs, a lentiviral construct was produced (Y-sh-SYNE1). **(B)** The down-regulation of SYNE1 was confirmed by real-time qPCR. *n* = 5/group; **P* < 0.05 Y-sh-SYNE1 vs. Y-c. Y-c: control lentivirus transduced Y hBM-MSCs.

Supplementary Figure 3 Down-regulation of SYNE1 in O-sh-CYP7A1 hBM-MSCs. O-sh-CYP7A1 hBM-MSCs were transfected by SYNE1 inhibition lentivirus (O-sh-CS) or control lentivirus (O-sh-CC), respectively. The down-regulation of SYNE1 was confirmed by real-time qPCR. *n* = 5/group; **P* < 0.05 O-sh-CC vs. O-c, ^#^*P* < 0.05 O-sh-CS vs. O-sh-CC.

Supplementary Figure 4 Lnc-CYP7A1-1 down-regulation increased old hBM-MSCs survival *in vivo.* The survival of the implanted cells was detected by green fluorescent protein (GFP) expression, which was carried out by the lentiviral-vector transduction prior to cell transplantation in the border region of the infarcted mouse hearts at 3 days post MI. *n* = 6/group; ^#^*P* < 0.05 O-sh-CYP7A1 vs. O-c.

Supplementary Figure 5 Down-regulation of lnc-CYP7A1-1 in O hBM-MSCs changed the cell paracrine function *in vitro*. Quantification of mRNA expression of VEGFA, PDGFA, FGF2, IGF1, TGFB1, ANG, and CCL2 in the O-c and O-sh-CYP7A hBM-MSCs after cultured for 72 h under hypoxia conditions by real-time qPCR. *n* = 6/group; ^#^*P* < 0.05 O-sh-CYP7A vs. O-c.

Supplementary Table 1 qRT-PCR primer sequences.

## Abbreviations

lncRNA: long non-coding RNA
hBM-MSCs: human bone marrow mesenchymal stem cells
CCK8: cell counting kit-8
MI: myocardial infarction
FS: fractional shortening
EF: ejection fraction
LVIDs: left ventricular internal end systolic dimension
LVIDd: left ventricular internal end-diastolic dimension.
