# Supplementary figures and images for: Down-Regulation of Lnc-CYP7A1-1 Rejuvenates Aged Human Mesenchymal Stem Cells to Improve Their Efficacy for Heart Repair Through SYNE1
Source: Front Cell Dev Biol. 2020 Nov 19;8:600304. doi: 10.3389/fcell.2020.600304 (PMC7710953; doi:10.3389/fcell.2020.600304)

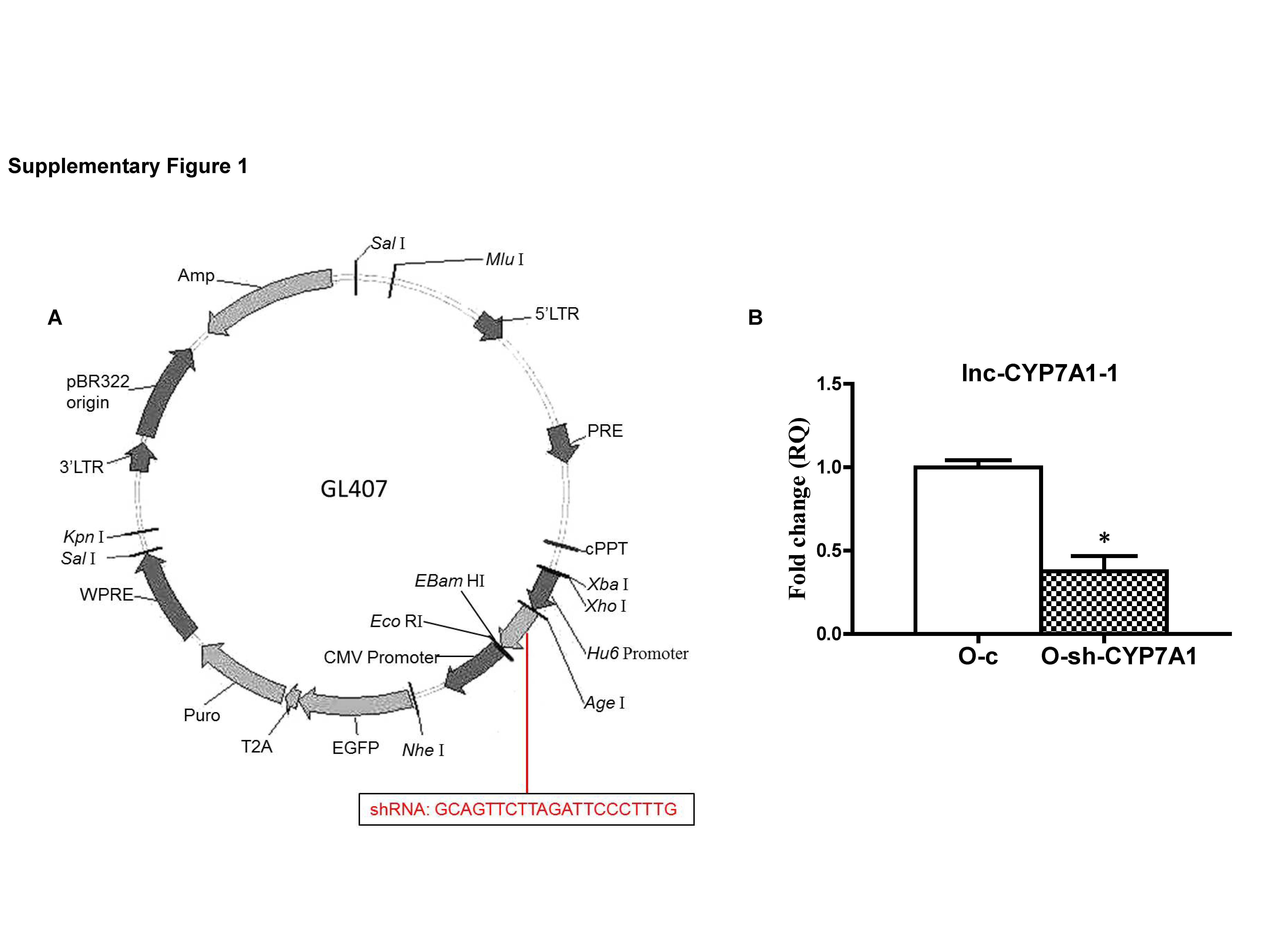

Supplement: Supplementary Figure 1 — The expression of lnc-CYP7A1-1 was inhibited by lentivirus. (A) To inhibit lnc-CYP7A1-1 expression in Old (O) hBM-MSCs, a lentiviral construct was produced (O-sh-CYP7A1). (B) The down-regulation of lnc-CYP7A1-1 was confirmed by real-time qPCR. n = 5/group; *P < 0.05 O-sh-CYP7A1 vs. O-c. O-c: control lentivirus transduced O hBM-MSCs. [file Image_1.TIFF]

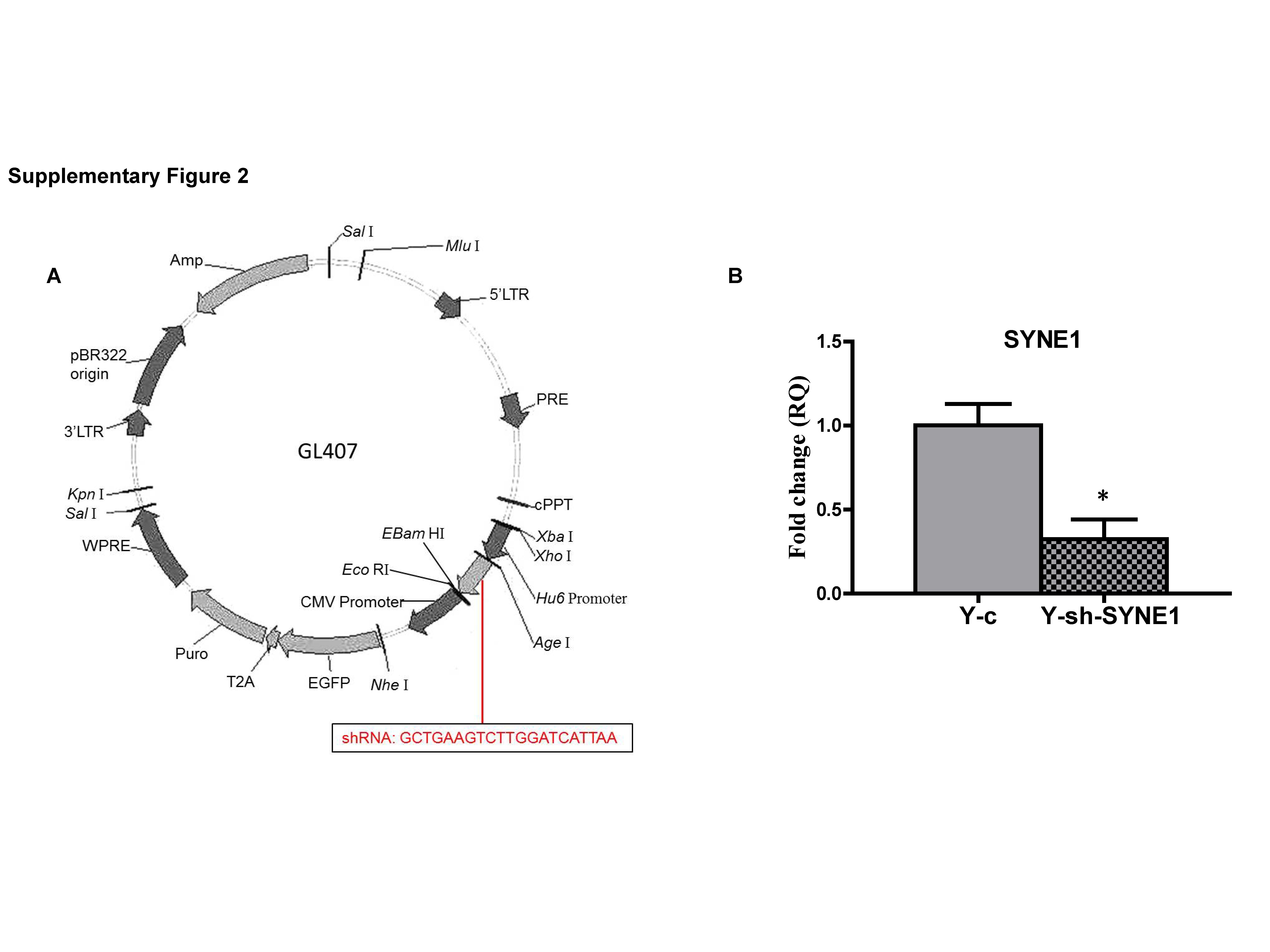

Supplement: Supplementary Figure 2 — The expression of SYNE1 was inhibited by lentivirus in Y hBM-MSCs. (A) To inhibit SYNE1 expression in young (Y) hBM-MSCs, a lentiviral construct was produced (Y-sh-SYNE1). (B) The down-regulation of SYNE1 was confirmed by real-time qPCR. n = 5/group; *P < 0.05 Y-sh-SYNE1 vs. Y-c. Y-c: control lentivirus transduced Y hBM-MSCs. [file Image_2.TIF]

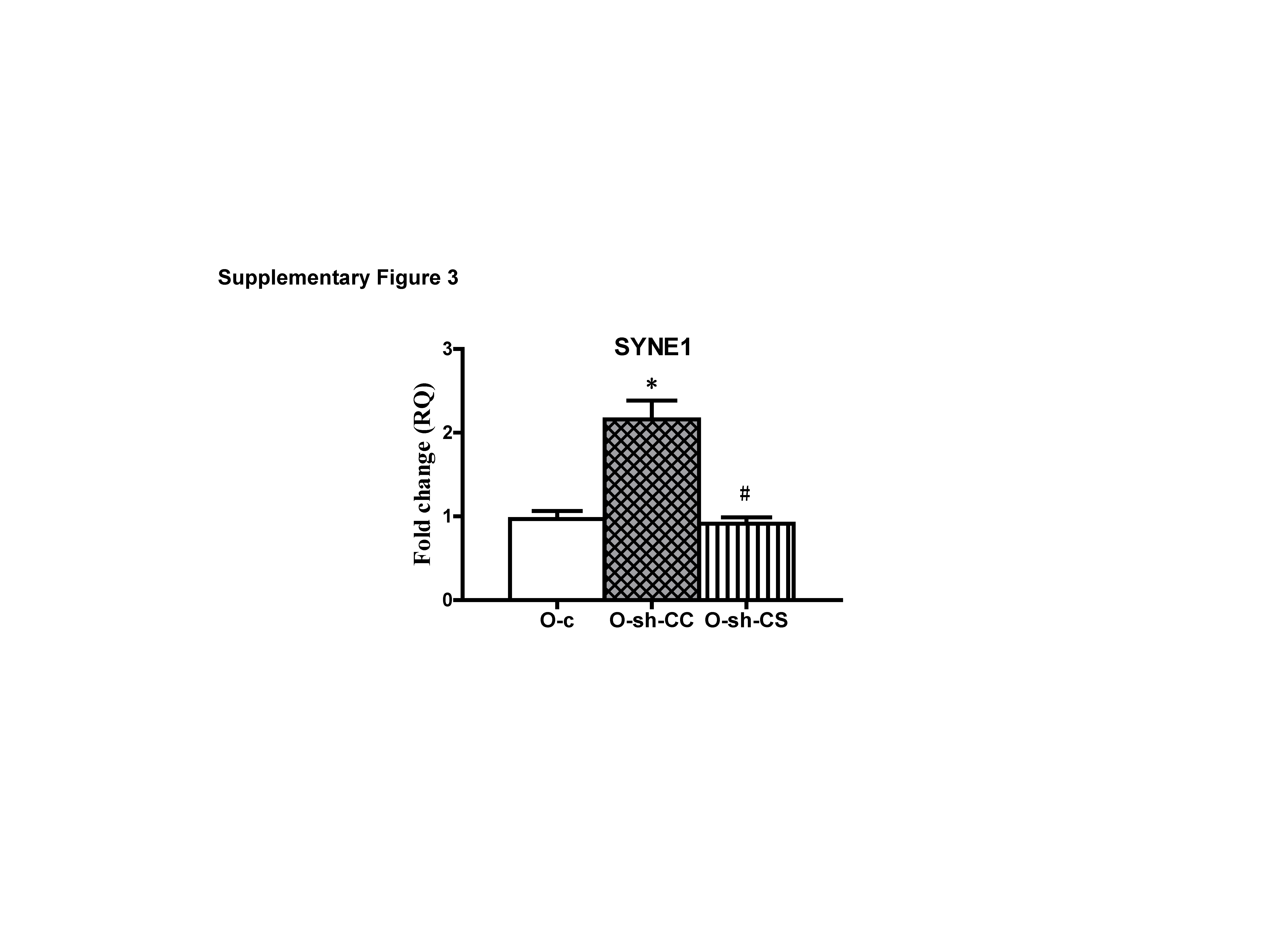

Supplement: Supplementary Figure 3 — Down-regulation of SYNE1 in O-sh-CYP7A1 hBM-MSCs. O-sh-CYP7A1 hBM-MSCs were transfected by SYNE1 inhibition lentivirus (O-sh-CS) or control lentivirus (O-sh-CC), respectively. The down-regulation of SYNE1 was confirmed by real-time qPCR. n = 5/group; *P < 0.05 O-sh-CC vs. O-c, #P < 0.05 O-sh-CS vs. O-sh-CC. [file Image_3.TIF]

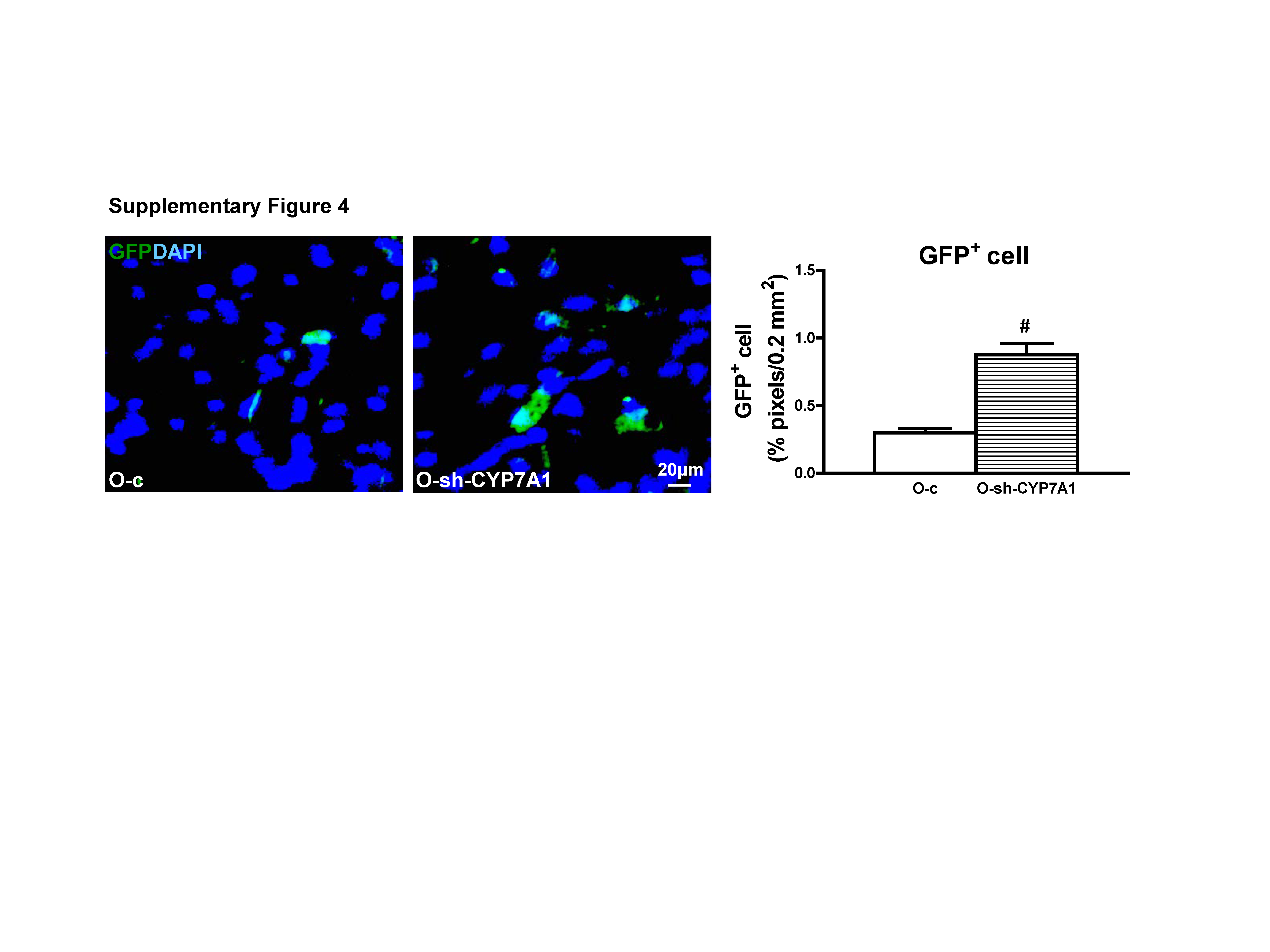

Supplement: Supplementary Figure 4 — Lnc-CYP7A1-1 down-regulation increased old hBM-MSCs survival in vivo. The survival of the implanted cells was detected by green fluorescent protein (GFP) expression, which was carried out by the lentiviral-vector transduction prior to cell transplantation in the border region of the infarcted mouse hearts at 3 days post MI. n = 6/group; #P < 0.05 O-sh-CYP7A1 vs. O-c. [file Image_4.TIFF]
