# Supplementary material for: Down-Regulation of Lnc-CYP7A1-1 Rejuvenates Aged Human Mesenchymal Stem Cells to Improve Their Efficacy for Heart Repair Through SYNE1
Source: Front Cell Dev Biol. 2020 Nov 19;8:600304. doi: 10.3389/fcell.2020.600304 (PMC7710953; doi:10.3389/fcell.2020.600304)
Supplement: Supplementary Table 1 — qRT-PCR primer sequences. [file Table_1.docx]

**Supplementary Table 1. qRT-PCR primer sequences.**

| **Name** | **Forward Primer** | **Reverse Primer** |
| --- | --- | --- |
| P16 | GATGTCGCACGGTACCTG | TCTCTGGTTCTTTCAATCGGG |
| P27 | TCTGAGGACACGCATTTGG | TGTTCTGTTGGCTCTTTTGTTT |
| LINC01366 | GTCTGTATGGTGGCTCGTGAAG | ACCTGCTGTCTGGGACTGATG |
| lnc-RBBP6-4 | CCTCTCCTTTGCCTTTGGTCT | TCTCCGGGCCTCAGTTTTC |
| SH3TC2-DT | TGGGTGACCAGGTGAAGAATG | GAAGGGAGGGAGTTGGACAAG |
| LINC01809 | ACACAGGCACAAAGCAGAAGC | TACCGGCACTGTGACATCCA |
| GAPDH | AACGGATTTGGTCGTATTG | GGAAGATGGTGATGGGATT |
| lnc-OR4F5-7 | AGACTGTCTGCTGGTGGGATG | CCTCTGCTGTATGTCGGGAAT |
| lnc-CYP7A1-1 | CAGCACTGGTAAAGGGAGGG | GGAGCAGGTGGAGAATGAGG |
| TRHDE-AS1 | TCTCCACCCTGGGCACTTTA | ATGAGGGAGTCTCGCTGTCG |
| lnc-MYO10-2 | CGGATTGATAACAGGGACAGC | TTCCTCACAACATCGCCTACAC |
| TNFRSF14-AS1 | AGCTTTGGACATCGGTTTGG | ATTAGGAGTGGAGTTGCTGGGT |
| LINC00222 | TCTGGCAAAGAGGAACTGGC | ACTCGTGGTCTGTGGGACATG |
| LINC02372 | AGTCGGCAGTAGAGGACCCAT | AGAGGAATCCACGCATCCAG |
| LINC02267  VEGFA  PDGFA  FGF2  IL6  IGF1  TGFB1  ANG  CCL2 | CAAGGCAGATGTGGGAAATGA  GATACCTCGCCCATGTTCTG  GATACCTCGCCCATGTTCTG  ACCCTCACATCAAGCTACAAC  CCACTCACCTCTTCAGAACG  GGAGGCTGGAGATGTATTGC  GCCTTTCCTGCTTCTCATGG  ACCCATCTCCAGGAACAAAC  CAGAAGTGGGTTCAGGATTCC | CCTTGTTAGCCAGGATGGTCTC  CAAAGAATCCTCACTCCCTACG  CAAAGAATCCTCACTCCCTACG  AAAAGAAACACTCATCCGTAACAC  CATCTTTGGAAGGTTCAGGTTG  TCCTGTAGTTCTTGTTTCCTGC  TCCTTGCGGAAGTCAATGTAC  CAGACCCAGCACGAAGAC  ATTCTTGGGTTGTGGAGTGAG |
